# Slurry Homopolymerization of Ethylene Using Thermostable α-Diimine Nickel Catalysts Covalently Linked to Silica Supports via Substituents on Acenaphthequinone-Backbone

**DOI:** 10.3390/polym14173684

**Published:** 2022-09-05

**Authors:** Kening Zong, Yanhui Hou, Xiaobei Zhao, Yali Sun, Binyuan Liu, Min Yang

**Affiliations:** 1 Hebei Key Laboratory of Functional Polymers, Institute of Polymer Science and Engineering, Hebei University of Technology, Tianjin 300130, China; 2 State Key Laboratory of Separation Membranes and Membrane Processes, School of Material Science and Engineering, Tiangong University, Tianjin 300160, China

**Keywords:** α-diimine nickel(II) catalyst, heterogeneous catalysis, branched polyethylene, silica support

## Abstract

Four supported α-diimine nickel(II) catalysts covalently linked to silica via hydroxyl functionality on α-diimine acenaphthequinone-backbone were prepared and used in slurry polymerizations of ethylene to produce branched polyethylenes. The catalytic activities of these still reached 10^6^ g/molNi·h at 70 °C. The life of the supported catalyst is prolonged, as can be seen from the kinetic profile. The molecular weight of the polyethylene obtained by the 955 silica gel supported catalyst was higher than that obtained by the 2408D silica gel supported catalyst. The melting points of polyethylene obtained by the supported catalysts S-C1-a/b are all above 110 °C. Compared with the homogeneous catalyst, the branching numbers of the polyethylenes obtained by the supported catalysts S-C1-a/b is significantly lower. The polyethylenes obtained by supported catalyst S-C1-a/b at 30–50 °C are free-flowing particles, which is obviously better than the rubber-like cluster polymer obtained from homogeneous catalyst.

## 1. Introduction

The polymerization of ethylene catalyzed by α-diimine complex is a major breakthrough in the field of polyolefin synthesis [1,2]. In recent decades, the polyolefin field has been keen to enrich the types of α-diimine complexes via introducing different substituents or changing the skeleton structure of α-diimine ligands, and it is true that these strategies could not only improve their activity and stability but also achieve pruning of the polymer microstructure [3,4,5,6,7,8,9,10,11,12,13,14,15,16,17,18,19,20,21]. However, it is challenging to use α-diimine catalysts in gas-phase and slurry commercial polymerization processes. The major disadvantages of homogeneous α-diimine catalysts are the shorter lifetimes in polymerization processes, especially the lack of control over the morphology of polymer particles, resulting in serious reactor fouling. In order to improve this problem and adapt to the polymerization process, the general method is to immobilize the homogeneous catalyst on a suitable support [22,23,24,25,26]. The SiO_2_ and MgCl_2_ are the commonly used supports.

**Scheme 1:**
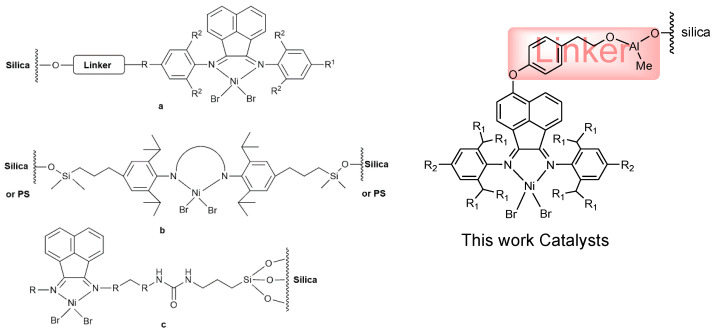
Supported α-diimine nickel(II) catalysts. (**a**): [27]; (**b**): [28]; (**c**): [29].

Although the ionic bond-supporting approach attaches the active center to the support, many of the catalysts obtained will still be leached, which has a certain negative effect on the active center. In contrast, the covalent bond-supporting rule can greatly alleviate the above defect. The typical approach of preparing supported catalyst involves, firstly, pretreatment of the support with an activator to form linking groups on the surface of the support. Then, the support is connected with the catalyst with special functional groups through covalent bonds. The covalent bond between α-diimine catalysts, with functional groups introduced, and the support avoids the elution of homogeneous catalysts in the process of polymerization, thus, producing polymer particles with good morphology. Brookhart’s group prepared a covalent bond-supported α-diimine nickel(II) catalyst via introducing hydroxyl or amino functional groups to the *p*-aryl position and connecting it with trimethylaluminum treated silica gel (Scheme 1a) [27]. Li’s group used Si-Cl end groups to modify the allyl-containing α-diimine nickel(II) ligand and fixed it by chemical reaction with Merrifield resin, modified via a modifier with silicone or ethanolamine functional groups (Scheme 1b) [28]. Although its activity only reached 3.42 × 10^5^ g/molNi·h at 70 °C, the supported catalyst possesses a high loading rate and good chemical stability, and the high molecular weightbranched polyethylene also largely avoids the fouling of the reactor. Kim’s group prepared the α-diimine catalyst with trialkoxysilane functional group and immobilized it on silica supports through covalent bonds to obtain supported catalysts (Scheme 1c) [29]. The highest activity of ethylene polymerization was 2.56 × 10^5^ g/molNi·h at 70 °C. Jiang’s group loaded the α-diimine catalyst onto SiO_2_-MgCl_2_ bisupports or MgCl_2_/AlR_n_(OEt)_3-n_ supports to prepare supported catalysts for the ethylene polymerization [30,31]. The MgCl_2_ and SiO_2_ were used as supports, respectively, to support the α-diimine nickel(II) catalyst by Chio’s group [32,33], and the catalyst supported by MgCl_2_ had higher activities, but, unfortunately, it was not as strong as that of the SiO_2_-based supported catalytic system. Sun’s group prepared different aluminum compound modified silica carriers to support 1-(2,6dibenzhydryl-4-nitrophenylimino)-2-mesityliminoacenaphthyl nickel bromide [34]. Compared with the homogeneous system, it was found that the polymer by supported catalysts was branched polyethylene with high molecular weight, spherical shape and moderate branching degree, which proved that these catalysts had great potential.

Besides, organic supports, molecular sieve supports, mesoporous silicas and nanochannels mesoporous silicas are used as supports for immobilizing α-diimine catalysts, each of which has its uniqueness [35,36,37,38,39].

From the results of the previous literature, it can be seen that the immobilization of α-diimine catalyst can not only prolong the life of the catalyst and stabilize the catalytic process but also avoid the pollution of the reaction device and the slow heat dissipation in the process of homogeneous polymerization. The particle morphology and processability of the polymer prepared by the supported catalyst were obviously better than those of the polymer prepared by the homogeneous catalyst system. However, when the polymerization temperature reached 70 °C, the activities of these supported catalysts decreased significantly, which were not suitable for use in industrial polymerization temperature. This phenomenon is mainly caused by the ligand structure of the catalyst itself. From the structure of reported supported α-diimine catalysts, the small steric hindrance of substituents on the ligand is the main reason for the poor thermal stability. Another possible reason is the effect of the supports on the catalytic active center; the support can act as a barrier for monomer and cocatalyst access. In the previous studies of supported α-diimine catalyst, the ligand was grafted onto the supports by chemical reaction of the activated support through the functional group carried on the para-position of aniline. According to the reported support grafting strategy of this kind of aniline, the support occupied the para-position of aniline, and its catalytic active center was close to the support, which made the larger steric hindrance of the support and the reactive groups on the surface of the support had a negative effect on the catalytic active center. Meanwhile, the occupancy of para-position aniline reduces the tunability of the N-aryl substituents.

**Scheme 2:**
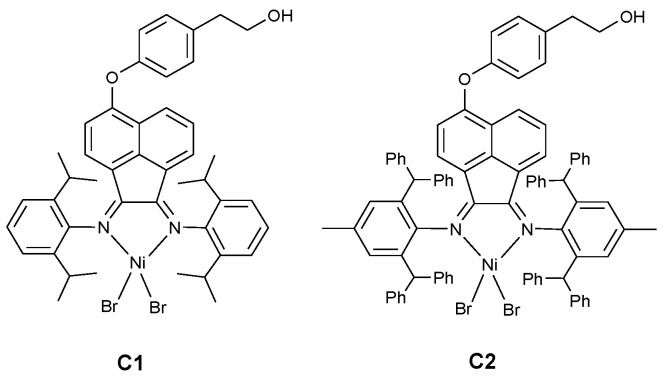
The α-diimine nickel(II) complexes.

So, we will attempt to prepare the novel covalent attachment supported α-diimine catalyst, which is formed by the reaction of the functional group on the side of the α-diimine acenaphthequinone backbone with the activated support. In this way, the active center of the catalyst can be far away from the support, to prevent or attenuate the effect of the support on the catalytic activity of the catalyst. At the same time, with the change of the N-aryl substituents, a large variety of supported α-diimine catalysts can be readily obtained, which greatly improves the tunability of the catalyst structure for the catalytic performance and the polymer structure. For example, the thermal stability of the catalyst can be improved via increasing the steric hindrance of the aniline substituent on the ligand. We have synthesized two α-diimine catalysts with hydroxyethyl phenoxyl group on acenaphthequinone-backbone (Scheme 2), which showed a higher catalytic activity and good heat resistance in ethylene polymerization [40]. In this work, these α-diimine catalysts were supported on silica gel by the reaction of Hydroxyl on acenaphthequinone backbone with the activated SiO_2_, and four kinds of supported catalysts were prepared for catalytic ethylene polymerization. Meanwhile, the effects of the types of silica gel, N-aryl substituents in ligand, reaction temperature on the catalytic activity, product branching degree, product melting point, product morphology and molecular weight and distribution of the product were investigated.

## 2. Materials and Methods

### 2.1. Materials

All the reactions sensitive to water and oxygen were performed under a purified nitrogen atmosphere using Schlenk technique. Diethylaluminum chloride (1 M solution in hexane) and trimethylaluminum (TMA, 1 M solution in hexane) were purchased from Yanfeng Technology Co., Ltd. (Shenyang, China). The α-diimine Ni(II) catalysts were prepared according to the literature (Scheme 2) [41]. Hexane solvent and toluene solvent were dried through soaking the active molecular sieve (4Å) then refluxed with sodium diphenylketyl in distillation unit prior to use. In addition, dichloromethane (CH_2_Cl_2_) were dried through calcium hydride. The silica gel (955 and 2408D) was supplied by Daqing Chemical Research Center. High-purity ethylene monomer was purchased from Air Products & Chemicals Co., Ltd. (TianJin, China). All other experimental supplies and chemicals were obtained commercially and used as received.

### 2.2. Characterization

Nickel loading of supported catalysts was characterized by inductively coupled plasma (ICP) atomic emission spectrometer on ICP-715ES (Thermo Jarrell Ash Co., Waltham, MA, USA). The morphology of supported catalysts, silica supports and polyethylenes were determined by scanning electron microscopy (SEM) on NovanosM 450 (FEI Co., Hillsboro, OR, USA). The pore size, surface area and pore volume of supported catalysts and silica supports were measured by using a standard Brunauer Emmett Teller (N_2_-BET) method performed by ASAP-2000 (Micromeritics Instrument Co., Norcross, GA, USA). The X-ray photoelectron spectroscopy (XPS) of supported catalyst was performed on Thermo Scientific ESCALAB Xi+ (Thermo Fisher Scientific, Waltham, MA, USA). Molecular weight and molecular weight distribution of branched polyethylenes were tested by PL-GPC-220 in 1,2,4-trichlorobenzene solution at 150 °C (Agilent Technologies, Inc., Santa Clara, CA, USA). The branched chain structure of polyethylene was tested on Bruker DMX 400 MHz instrument and ^13^C NMR spectra was derived, in which 1,2-dichlorobenzene was used as the internal standard and the temperature was 120 °C (Bruker Co., Billerica, MA, USA). The fusion enthalpy (Δ*H*_f_) and melting temperature (*T*_m_) of branched polyethylenes were characterized by differential scanning calorimetry (DSC) with DSC-Diamond (PerkinElmer, Inc., Waltham, MA, USA). In nitrogen atmosphere, the temperature range of the instrument was 0 °C to 150, and the heating rate was 10 °C/min. Using the melting temperature measured during the second heating up, the crystallinity (*χ*_c_%) was calculated by the formula (Δ*H*_f_/Δ*H*_f_°) × 100%, where Δ*H*_f_° is the melting heat of folded chain polyethylene (289.0 J/g) [42].

### 2.3. Synthesis of Supported α-Diimine Nickel(II) Catalysts

The mechanical stirring device was assembled, and silica gel (2 g, 955 or 2408D, calcined at 200 °C) and 50 mL of distilled toluene solvent were successively added, then 6 mL AlMe_3_ (1 M, hexane solution) was added at 0 °C, and the device was left to rest for a while before stirring. After heating up to 40 °C and stirring for 6 h, the excess solution was removed, and then the activated silica supports were washed with hexane and toluene, respectively. Activated silica gel was filtered and dried in vacuum for 10 h at 20 °C. Under mechanical stirring, activated silica supports were soaked in CH_2_Cl_2_, and the solution of the α-diimine Ni(II) catalysts in CH_2_Cl_2_ was added at 40 °C. After stirring for 8 h, the supported catalysts were washed with CH_2_Cl_2_ for many times and dried in vacuum under 20 °C for 10 h. According to this method, the supported α-diimine Ni(II) complexes S-C1-a, S-C2-a, S-C1-b, S-C2-b were prepared, respectively. In the names of the catalysts, S represents the support, C1 or C2 is homogeneous catalyst in Scheme 2, a and b represent the support 955 silica gel and 2408D silica gel, respectively.

### 2.4. Polymerization

It is worth noting that the 100 mL reactor needed to be baked in a constant temperature drying box at 100 °C for 6 h before assembly, then purged with argon for three times and injected ethylene gas to maintain the ethylene atmosphere in the device. The reactor was kept at the required polymerization temperature, and then a certain amount of solvent, co-catalyst solution and catalyst were added sequentially, and ethylene gas was injected to carry out the ethylene polymerization for 60 min. After the polymerization was stopped, the reactor was cooled to room temperature and the polymerization mixture was quenched by addition of 10 vol% HCl/ethanol solution. Then, the polyethylenes were washed with anhydrous ethanol and distilled water and then baked 8 h to 12 h in a 60 °C vacuum oven to achieve constant weight.

## 3. Results and Discussion

### 3.1. Synthesis and Characterization of Supported Catalysts

**Scheme 3:**
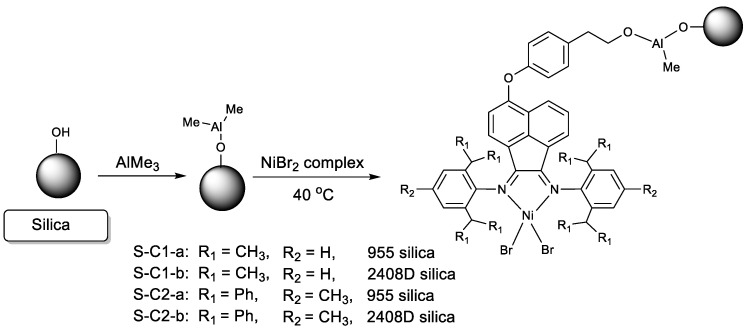
Preparation of silica-supported α-diimine nickel(II) catalysts.

Two types of silica gel (955 and 2408D) were not treated according to the traditional method of calcining above 600 °C for a long time, instead drying the adsorbed moisture at 200 °C, and then being treated with TMA. Subsequently, covalent attachment (Scheme 3) was obtained by chemical reaction of hydroxyl groups of α-diimine nickel(II) catalysts (C1 and C2, Scheme 2) with alumina organic compounds on activated silica gel. The supported α-diimine Ni(II) complexes S-C1-a, S-C2-a, S-C1-b, S-C2-b were prepared, respectively.

**Figure 1:**
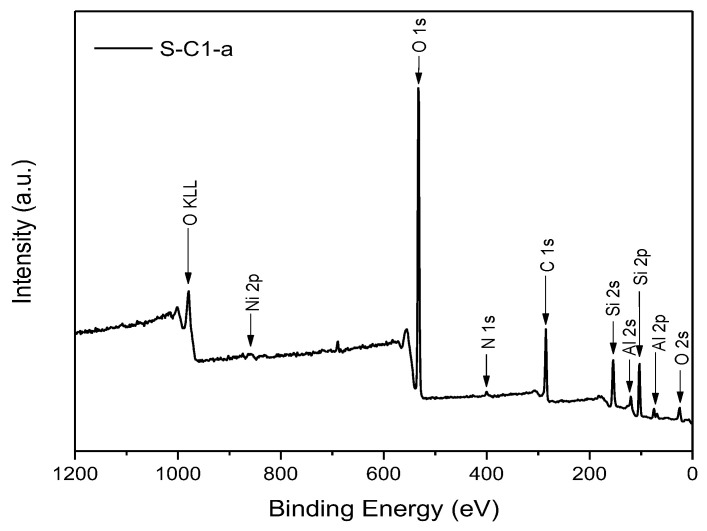
XPS wide scan spectrum of covalently supported catalyst S-C1-a.

**Figure 2:**
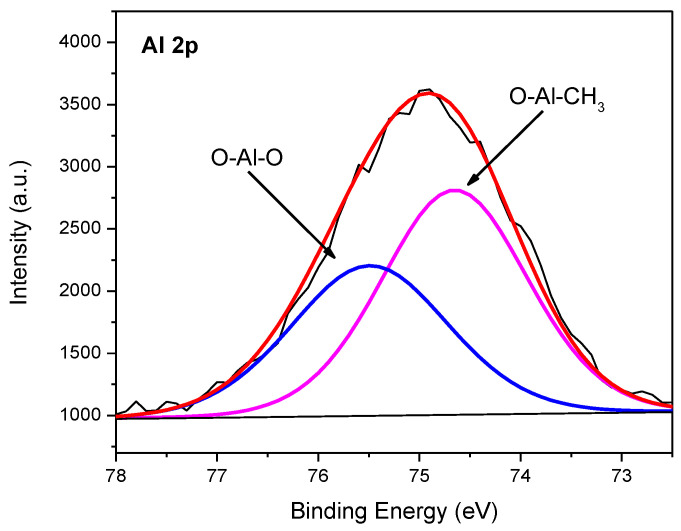
Al 2p level spectra of covalently supported catalyst S-C1-a.

In order to further explore the specific grafting mode between the homogeneous catalysts and supports, we took the supported catalyst S-C1-a as an example to carry out X-ray photoelectron spectroscopy characterization, and the results are shown in Figure 1 and Figure 2. The peaks at binding energy (BE) of the supported catalyst were about 285, 400, 75, 859, 102 and 533 eV, which were attributed to C 1s, N 1s, Al 2p, Ni 2p^3^, Si 2p and O 1s. For SiO_2_-supported catalysts, the absorption signals of Si 2p and O 1s were very strong, which was related to the large amount of silicon and oxygen on the surface of SiO_2_. In addition, the Al element in the activated supports is an important connection element between the homogeneous catalyst and the supports. Figure 2 shows the Al 2p level spectrum of covalently supported catalyst. It can be seen that the absorption of Al 2p occurs at approximately 75.5 eV and 74.7 eV, which were attributed to O-Al-O and O-Al-CH_3_, respectively [43]. Among them, the absorption peak of O-Al-O at 75.6 eV indicated that the α-diimine nickel(II) catalyst had been successfully grafted to SiO_2_ through the covalent bond.

Nickel contents in the covalently supported catalysts were identified by ICP analysis. The theoretical nickel loading of C1 on 955 and 2408D silica gel was 1.40 wt%, and the measured nickel loading of C1 on 955 and 2408D silica gel was 1.25 wt% and 1.06 wt%, respectively, which was only slightly lower than the theoretical maximum nickel loading. However, the measured nickel loading of C2 on 955 and 2408D silica gel was 0.54 wt% and 0.53 wt%, respectively, which were almost reduced to half of the theoretical nickel contents (1.20 wt%). These results are related to the steric hindrance of the catalyst. Compared to the steric hindrance of isopropyl substituents on C1, the steric hindrance of diphenylmethyl substituents at the C2 N-aryl position is significantly greater. The large steric hindrance of C2 hinders the reaction of the hydroxyl group of the catalysts with the active supports, so the nickel content was significantly reduced. Here, we report the catalytic activity per mole of the catalyst based on the detected nickel content. Furthermore, the surface area and pore structure of silica gel and supported catalysts were analyzed by BET, and the data were summarized in Table 1.

Although the specific surface area of 2408D silica gel is higher than that of 955 silica gel, the pore size and pore volume of 2408D silica gel are less than that of 955 silica gel. The BET data show that pore size, specific surface area, and pore volume of the silica-supported catalysts are smaller than those of the supports. This indicates that the catalyst was successfully supported on the surface of the silica gel and in the pores. After loading on the same silica gel, the specific surface area of the C2-supported catalyst decreases less than that of the C1-supported catalyst, which is consistent with the large steric hindrance and low nickel loading of C2 catalyst. The catalytic properties of the catalyst and the properties of the polyethylenes are restricted by various factors of the support and the supported catalyst.

### 3.2. Ethylene Polymerization

Ethylene slurry polymerizations were carried out by using supported catalysts S-C1-a/b and S-C2-a/b in hexane with AlEt_2_Cl, and the supported catalysts were obtained by loading catalyst C1 (or C2) on two different silica gels. The ethylene polymerization results were summarized in Table 2 and Table 3. The kinetic distributions of ethylene polymerization catalyzed by the supported catalyst S-C1-a and homogeneous catalyst C1 are shown in Figure 1. We found that the kinetic of the homogeneous catalyst C1 is a typical decay type, with a high initial rate and then rapid decay, the polymerization was basically stopped in about 30 min. Although the catalyst C1 displays higher activity than supported system S-C1-a at the initial stage, the catalytic activity decay rate (or deactivation rate) of the catalyst C1 is much faster than that of the catalyst S-C1-a. We believe that the introduction of spherical silica gel into the homogeneous catalysts not only obstructs the insertion of ethylene, decelerating the rate of association replacement, but also reduces the deactivation rate of the catalysts and prolongs the life of the catalysts.

**Figure 3:**
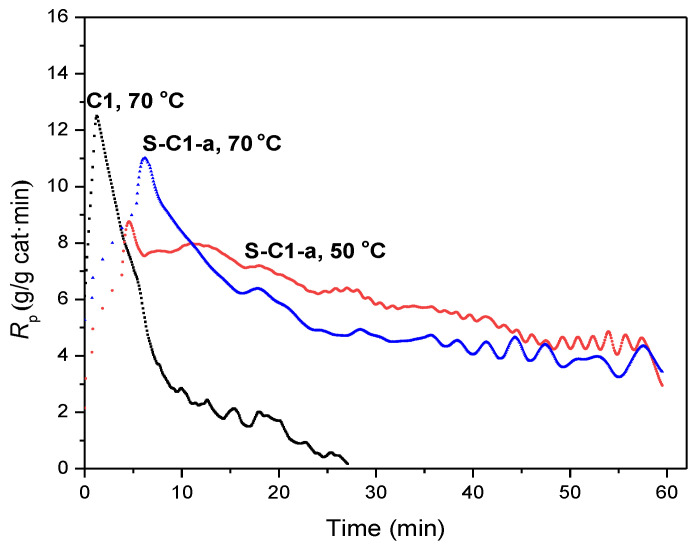
The kinetic curves of ethylene polymerization.

Temperature inevitably has an influence on the catalytic activity and the molecular weight of the resultant polyethylene. At elevated temperatures, the catalytic activity increased firstly and then decreased. When the polymerization temperature was 50 °C, the kinetic profile (Figure 3) of ethylene polymerization was almost stable. However, with the increase in temperature up to 70 °C, the initial stage exhibited a higher reaction rate, but with the extension of polymerization time, the activity decreased gradually.

However, the catalytic activities of four supported catalysts still reached 10^6^ g/molNi·h at 70 °C and 0.5 MPa ethylene pressure, which suggested that these supported catalysts had good thermal stability. The molecular weight of the resultant polyethylene decreased with the increase in temperature. For example, the molecular weight decreased from 774 kg/mol at 30 °C to 261 kg/mol at 70 °C for catalyst S-C1-b (Entries 5 and 7, Table 2). This phenomenon may be caused by the acceleration of chain transfer at high temperature.

In the experiment, we used two different kinds of silica gel to prepare supported catalysts. From the data in the two tables, it can be found that the two different supports have little influence on polymerization activity; the catalytic activity of the supported catalyst loaded by 955 silica gel is slightly higher than that of 2408D silica gel, but they have a great influence on the molecular weight of the obtained polymer. Previous studies have shown that it is not that the greater the specific surface area of the support, the greater the catalyst activity. Although the specific surface area of 955 silica gel was smaller than that of 2408D, the activity of the catalyst supported on 955 silica gel (S-C1-a/S-C2-a) was slightly higher than that of the catalyst supported on 2408D silica gel (S-C1-b/S-C2-b) at the same polymerization condition. The polyethylene obtained from catalyst S-C1-a (or S-C2-a) showed a higher molecular weight than that of the polyethylene obtained from catalyst S-C1-b (or S-C2-b) under same conditions. These phenomena may be related to the larger pore volume and pore size of 955 silica gel, and the higher nickel-loading rate of 955 supported catalysts. These factors are favorable for the polymerization of the monomer. For the catalysts supported on 2408D silica gel, lower nickel content makes the unloaded part of silica gel have a great negative effect on the loaded active center, which further affects the chain growth reaction.

Furthermore, we studied the cases of catalysts with different structures supported on the same silica gel. The substituents on N-aryl of complex C1 are 2,6-diisopropyl substituents, those of catalyst C2 are 2,6-diphenylmethyl substituents. Compared to catalysts S-C1-a/b, the activities of S-C2-a/b were lower than those of S-C1-a/b at the same polymerization condition. It should be related to the large steric hindrance with dibenzhydryl substituents of catalysts S-C2-a/b, which hinders the insertion speed of ethylene and resulting in the reduced activity at 30–50 °C. On the contrary, the large steric hindrance substituents on S-C2-a/b can reduce the occurrence of chain transfer during polymerization at high temperatures, and thus they showed the greater thermal stability. When the polymerization temperature further increases to 70 °C, the polymerization activity decreases slightly.

**Figure 4:**
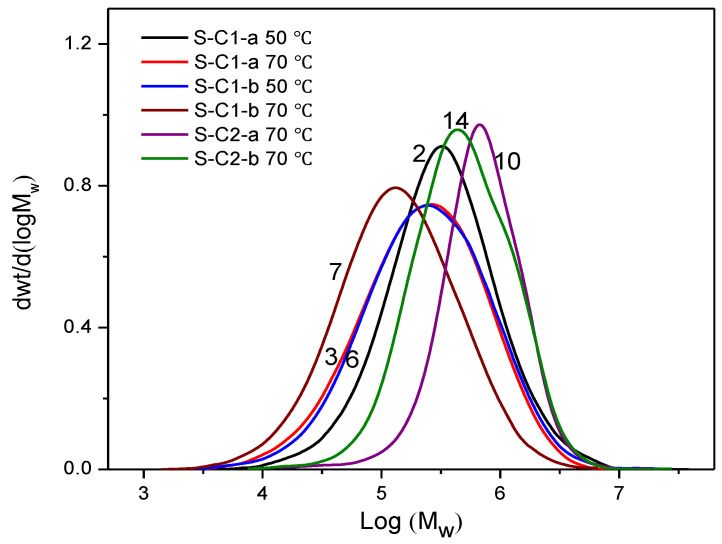
GPC curves of polyethylene obtained with S-C1-a/b, S-C2-a/b (Entries 2, 3, 6, 7 in Table 2, Entries 10, 14 in Table 3).

The structure of the catalyst also has a great influence on molecular weight of the obtained polymer. The polyethylenes obtained by catalysts S-C2-a/b performed the higher molecular weight of 911 kg/mol and 725 kg/mol (Entries 10 and 14, Table 3), which is more than two times as much as that of the polyethylenes prepared from catalysts S-C1-a/b (Entries 3 and 7, Table 2) under same conditions (Figure 4). On the other hand, S-C2-a/b reduces the polymerization rate due to the introduction of diphenyl with large steric hindrance, but diphenyl also effectively hinders the chain transfer reaction, which promotes the chain growth reaction and obtains high molecular weight polyethylenes. Other GPC curves of polyethylene are shown in the Supplementary Materials (Figures S1 and S2).

**Figure 5:**
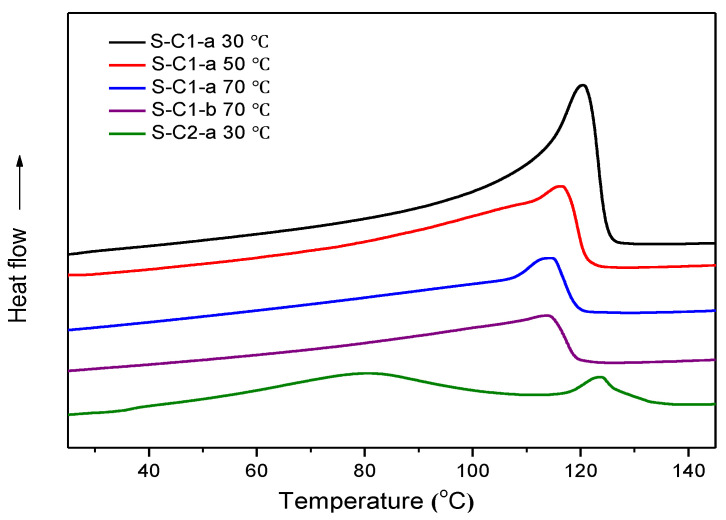
DSC curves of polyethylene obtained with S-C1-a/b, S-C2-a (Entries 1, 2, 3, 7 in Table 2, Entry 8 in Table 3).

The polyethylenes are determined by differential scanning calorimetry (DSC). The melting temperature (*T_m_*) and crystallinity data of the polyethylenes are summarized in Table 2 and Table 3. The DSC curves are in the Figures S3–S6 of the Supporting Material. Compared with the polyethylene obtained by the homogeneous catalyst C1 [40], which has low or even no melting point, the polyethylenes obtained by the supported catalysts S-C1-a/b have higher melting points and are above 110 °C, due to the inhibition of the support to the chain walking reaction during the polymerization process. Following the same trend as other reported α-diimine nickel(II) catalysts, the melting point of the obtained polyethylenes decreased with the increased polymerization temperature (Figure 5). This is due to the acceleration of chain growth and chain walking at higher temperatures, resulting in the formation of more branches that hinder the crystallization of the polymer. No significant influence of different silica gel on the melting point of resultant polyethylene under the same polymerization condition for the S-C1-a/b catalytic systems. At 70 °C polymerization temperature, the melting point of polyethylene obtained by S-C1-a is 114 °C, and that of polyethylene obtained by S-C1-b is 113 °C.

However, it is found that the melting points of polyethylene obtained by S-C2-a/b catalysts are close to those of homogeneous catalyst except the sample of Entry 8 in Table 3. We initially thought that although catalyst C2 was loaded on the support, these active sites leached from the silica support during the polymerization process. The large steric hindrance of the 2,6-dibenzhydryl substituents and the low relative proportion of the hydroxyl group with the large molecular weight of the catalyst further affect the reaction between the hydroxyl group of the catalyst and the modified silica gel to form a covalent bond, which makes the catalyst leaching off during the polymerization process. For the polymer of Entry 8 in Table 3, the two melting points behavior shows that the prepared polyethylene was a mixture of two chain populations with different chain structures, that is, there are two types of active centers in the polymerization system: those leached from the silica gel during polymerization and those kept fixed on the surface of the support.

**Figure 6:**
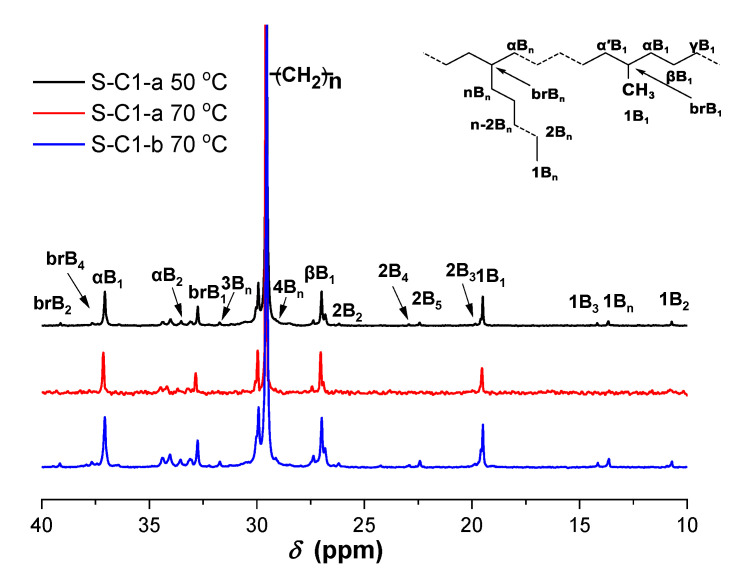
^13^C NMR spectra of polyethylenes (Entries 2, 3, 7 in Table 2).

The difference in melting point is mainly determined by the chain structure of the polymer, and the chain structures of the polymers were further characterized by the high-temperature ^13^C NMR spectroscopy. As shown in Figure 6, Table 2 and Figures S7–S9 of the Supporting Material being interpreted according to the literature [42], the polyethylene obtained by S-C1-a at 50 °C possessed 67/1000C branches, including methyl (64.4%), ethyl (16.7%), propyl (3.1%), butyl (2.7%), amyl (2.9%) and LCB (10.2%), while the polyethylene obtained by homogeneous catalyst C1 had high branching density, which is close to 100 branches/1000C [40]. Compared with the homogeneous catalyst, the branching numbers of the polyethylenes obtained by the supported catalysts S-C1-a/b is significantly lower. This may be due to the steric hindrance from the support, which hinders the “chain walking” rate significantly. Thus, the polyethylenes obtained by silica supported catalysts S-C1-a/b display higher melting temperatures. The branching degree of polyethylene obtained by S-C2-b catalyst is 63/1000C, which is close to that of the polyethylene catalyzed by homogeneous catalyst due to the leaching of the α-diimine catalyst C2 from the silica support catalyst. This result is consistent with the result of melting point.

As shown in Figure 6, when the temperature increased from 50 °C to 70 °C, the branching degree of polyethylene prepared using the catalyst S-C1-a increased from 67/1000C to 78/1000C while the resonances of methyl branch chain increased. Meanwhile, other branch chains decreased as the temperature increased. At the same polymerization temperature of 70 °C, although the branching density of polyethylenes obtained from S-C1-b (82/1000C) is slightly higher than that of S-C1-a, the resonances of other branch chains except methyl chain increased significantly. This phenomenon may be related to the difference of the support type, specific surface area and nickel content of the two supported catalysts, which greatly affects the mechanism of chain walking.

**Figure 7:**
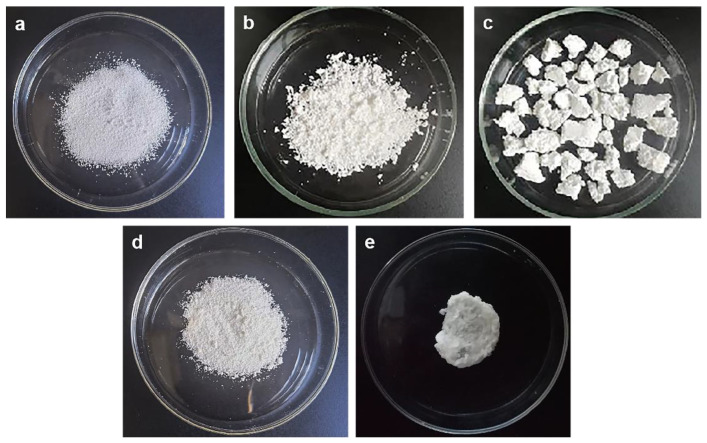
Photographs of polyethylenes obtained with ((**a**), S-C1-a 30 °C; (**b**), S-C1-a 50 °C; (**c**), S-C1-a 70 °C; (**d**), S-C1-b 30 °C; (**e**), homogeneous catalyst C1 30 °C).

**Figure 8:**
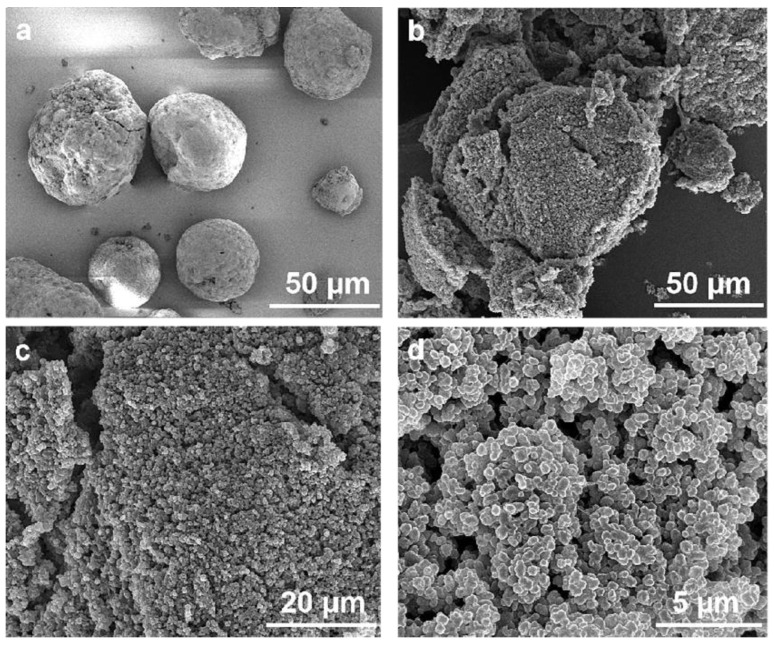
(**a**) SEM image of catalyst S-C1-a; (**b**) SEM image of 50 µm scale of polyethylene obtained with S-C1-a; (**c**) SEM image of 20 µm scale of polyethylene obtained with S-C1-a; (**d**) SEM image of 5 µm scale of polyethylene obtained with S-C1-a. (Entry 1, Table 2).

The morphology photos of the polymers and the SEM images of catalyst and the polymers are shown in Figure 7 and Figure 8. The polyethylene obtained by homogeneous catalyst C1 behaved like a rubber-like cluster (Figure 7e). Figure 7a,d show the free-flowing PE particles obtained with supported catalyst S-C1-a (or S-C1-b) at 30 °C (Entries 1 and 5, Table 2). The particle morphology of the polymer is obviously better than that obtained from the homogeneous system. When the polymerization temperature is raised to 50 °C, the polymer is slightly less dispersed and easy to accumulate together. Further increase the temperature up to 70 °C inevitably led to the formation of big shapeless polymer particles, due to the increase in branching density or part of the active component leaching (Figure 7c). Under the same polymerization conditions, the viscosity of polyethylene particles prepared by S-C2-a/b is higher than that of polyethylene particles prepared by S-C1-a/b, which may be due to the leaching of the α-diimine catalyst from the silica support catalyst caused by the steric hindrance of C2, and thus the morphology of polymer is very poor.

Furthermore, SEM was used to observe the microscopic morphology of the catalyst and polymer. In general, when the ethylene contacts the metal center on the support, polyethylene can be prepared and the resulting polyethylene will cover the support to replicate the shape of the support. In fact, the morphology control of the polymer particles is not only caused by the support but also by its fragmentation and the resulting distribution of active catalyst components. The silica support is filled with irregularly distributed pores, allowing α-diimine catalysts and monomers to penetrate silica gel to its inner surface. Therefore, the resulting polyethylene will cause the fragmentation of the support and no longer maintain the shape of the original catalyst particle. We can see this phenomenon in Figure 8a. Further magnification shows the accumulation of roughly spherical polymer subparticles with diameters of ~0.5 μm (Figure 8d).

## 4. Conclusions

α-Diimine catalysts with hydroxyethyl phenoxyl group on α-diimine acenaphthequinone-backbone were supported on two different silica gels by the reaction of hydroxyl groups on ligand backbone with the activated silica gel. These supported catalysts with AlEt_2_Cl cocatalyst afforded significant activity of ethylene polymerization. They had good thermal stability and the catalytic activities still reached 10^6^ g/molNi·h at 70 °C and 0.5 MPa ethylene pressure. The life of the catalyst is prolonged, seeing as the kinetic profile and the particle morphology of the polymer are obviously better than that obtained from the homogeneous system. Although the two different supports have little influence on polymerization activity, they have a great influence on the molecular weight of the obtained polymer due to the pore volume, pore size and nickel-loading rate. The melting points of polyethylenes obtained by the supported catalysts S-C1-a/b with 2,6-diisopropyl substituents on N-aryl are all above 110 °C. The branching numbers of the polymers obtained by S-C1-a/b are significantly lower than those obtained by the homogenous catalyst. However, for the supported catalysts S-C2-a/b, the catalyst was leached from the support during the polymerization process due to the excessive steric hindrance of dibenzhydryl substituents on the catalyst. Therefore, this kind of catalyst with larger steric hindrance substituents needs to adopt other methods to improve the supported effect. The further exploration of the supported catalyst is in progress. In general, the functionalization of the acenaphthequinone with a terminal hydroxyl groups proved to be a suitable choice for the synthesis of functionalized α-diimine catalysts. With the variation of N-aryl substituents, a variety of supported α-diimine catalysts can be easily obtained.

## Figures and Tables

**Table 1:** Physical properties of silica gel or supported catalyst.

Silica Gel or Supported Catalyst	BET Surface Area (m^2^/g)	Pore Volume (cm^3^/g)	Average Pore Diameter (nm)	Nickel Content (%)
955	280	1.50	18.20	-
2408D	318	1.10	15.10	-
S-C1-a	158	1.00	16.40	1.25
S-C1-b	220	0.90	14.10	1.06
S-C2-a	206	0.90	16.40	0.54
S-C2-b	212	0.80	14.20	0.53

**Table 2:** Results of ethylene polymerization catalyzed by S-C1-a/b catalysts ^a^.

Entry	Catalyst	*T* (°C)	A_1_^b^	A_2_^c^	*T*_m_^d^ (°C)	*χ*_c_^d^ (%)	*M*_w_^e^ (kg/mol)	PDI ^e^	Branches ^f^ (1000 C)
1	S-C1-a	30	1.64	349	120	33	n.d.	n.d.	n.d.
2	S-C1-a	50	2.08	443	116	31	563	3.9	67
3	S-C1-a	70	1.11	236	114	16	417	4.0	78
4	S-C1-b	10	0.24	44	123	56	n.d.	n.d.	n.d.
5	S-C1-b	30	1.42	258	118	38	774	4.0	56
6	S-C1-b	50	1.83	333	116	22	482	4.2	n.d.
7	S-C1-b	70	1.00	182	113	13	261	3.2	82

^a^ Conditions: P = 0.5 MPa, V(hexane) = 50 mL, n(Al)/n(Ni) = 1000. ^b^ Activity, 10^6^ gPE/(molNi·h). ^c^ Activity gPE/(gcat·h). ^d^ Determined by DSC. ^e^ Determined by GPC. ^f^ Determined by ^13^C-NMR. n.d. = not determined.

**Table 3:** Results of ethylene polymerization catalyzed by S-C2-a/b catalysts ^a^.

Entry	Catalyst	*T* (°C)	A_1_^b^	A_2_^c^	*T*_m_^d^ (°C)	*χ*_c_^d^ (%)	*M*_w_^e^ (kg/mol)	PDI ^e^
8	S-C2-a	30	1.02	94	80/123	16	n.d.	n.d.
9	S-C2-a	50	1.28	107	74	13	969	2.5
10	S-C2-a	70	1.19	109	60	15	911	2.3
11	S-C2-a	80	0.90	82	58	13	n.d.	n.d.
12	S-C2-b	30	0.87	78	79	20	n.d.	n.d.
13	S-C2-b	50	1.07	96	75	14	825	2.8
14	S-C2-b	70	1.01	91	61	11	725	2.5

^a^ Conditions: P = 0.5 MPa, V(hexane) = 50 mL, cocat: DEAC, n(Al)/n(Ni) = 1000. ^b^ Activity, 10^6^ gPE/(molNi·h). ^c^ Activity gPE/(gcat·h). ^d^ Determined by DSC. ^e^ Determined by GPC. n.d. = not determined.

## Data Availability

The authors declare data availability.

## Supplementary Materials

The following supporting information can be downloaded at: https://www.mdpi.com/article/10.3390/polym14173684/s1, Figure S1. GPC curves of polyethylene obtained with S-C1-a/b (Entries 2, 3, 5, 6, 7 in Table 2); Figure S2. GPC curves of polyethylene obtained with S-C2-a/b (Entries 9, 10, 13, 14 in Table 2); Figure S3. DSC curves of polyethylene obtained with S-C1-a (Entries 1–3 in Table 2); Figure S4. DSC curves of polyethylene obtained with S-C1-b (Entries 4–7 in Table 2); Figure S5. DSC curves of polyethylene obtained with S-C2-a (Entries 8–11 in Table 3); Figure S6. DSC curves of polyethylene obtained with S-C2-b (Entries 12–14 in Table 3); Figure S7. ^13^C NMR spectra of polyethylenes (Entries 2, 3 in Table 2); Figure S8. ^13^C NMR spectra of polyethylenes (Entries 5, 7 in Table 2); Figure S9. ^13^C NMR spectra of polyethylenes (Entries 13, 14 in Table 3).

## References

[1] Johnson L.K., Killian C.M., Brookhart M. (1995). New Pd(Ⅱ)- and Ni(Ⅱ)-based catalysts for polymerization of ethylene and a-olefins. J. Am. Chem. Soc..

[2] Small B.L., Brookhart M., Bennett A.M.A. (1998). Highly Active Iron and Cobalt Catalysts for the Polymerization of Ethylene. J. Am. Chem. Soc..

[3] Ittel S.D., Johnson L.K., Brookhart M. (2000). Late-Metal Catalysts for Ethylene Homo- and Copolymerization. Chem. Rev..

[4] AlObaidi F., Ye Z., Zhu S. (2004). Ethylene polymerization with homogeneous nickel–diimine catalysts: Effects of catalyst structure and polymerization conditions on catalyst activity and polymer properties. Polymer.

[5] Song C.-L., Tang L.-M., Li Y.-G., Li X.-F., Chen J., Li Y.-S. (2006). Preparation of linear α-olefins to high-molecular weight polyethylenes using cationic α-diimine nickel(II) complexes containing chloro-substituted ligands. J. Polym. Sci. Polym. Chem..

[6] Meinhard D., Wegner M., Kipiani G., Hearley A., Reuter P., Fischer S., Marti A.O., Rieger B. (2007). New Nickel(II) Diimine Complexes and the Control of Polyethylene Microstructure by Catalyst Design. J. Am. Chem. Soc..

[7] Bahuleyan B.K., Son G.W., Park D.-W., Ha C.-S., Kim I. (2008). Ethylene polymerization by sterically and electronically modulated Ni(II) α-diimine complexes. J. Polym. Sci. Part A Polym. Chem..

[8] Liu F.-S., Hu H.-B., Xu Y., Guo L.-H., Zai S.-B., Song K.-M., Gao H.-Y., Zhang L., Zhu F.-M., Wu Q. (2009). Thermostable α-Diimine Nickel(II) Catalyst for Ethylene Polymerization: Effects of the Substituted Backbone Structure on Catalytic Properties and Branching Structure of Polyethylene. Macromolecules.

[9] Popeney C.S., Guan Z. (2010). Effect of Ligand Electronics on the Stability and Chain Transfer Rates of Substituted Pd(II) α-Diimine Catalysts. Macromolecules.

[10] Rhinehart J.L., Mitchell N.E., Long B.K. (2014). Enhancing α-Diimine Catalysts for High-Temperature Ethylene Polymerization. ACS Catal..

[11] Du S., Kong S., Shi Q., Mao J., Guo C.-Y., Yi J., Liang T., Sun W.-H. (2015). Enhancing the Activity and Thermal Stability of Nickel Complex Precatalysts Using 1-[2,6-Bis(bis(4-fluorophenyl)methyl)-4-methyl phenylimino]-2-aryliminoacenaphthylene Derivatives. Organometallics.

[12] Guo L., Dai S., Chen C. (2016). Investigations of the Ligand Electronic Effects on α-Diimine Nickel(II) Catalyzed Ethylene Polymerization. Polymers.

[13] Wang Z., Liu Q., Solanad G.A., Sun W.-H. (2017). Recent advances in Ni-mediated ethylene chain growth: Nimine-donor ligand effects on catalytic activity, thermal stability and oligo-/polymer structure. Coord. Chem. Rev..

[14] Wu Z., Hong C., Du H., Pang W., Chen C. (2017). Influence of Ligand Backbone Structure and Connectivity on the Properties of Phosphine-Sulfonate Pd(II)/Ni(II) Catalysts. Polymers.

[15] He F., Wang D., Jiang B., Zhang Z., Cheng Z., Fu Z., Zhang Q., Fan Z. (2018). Introducing electron-donating substituents on ligand backbone of α-diimine nickel complex and the effects on catalyst thermal stability in ethylene polymerization. Inorg. Chim. Acta.

[16] Brown L.A., Anderson W.C., Mitchell N.E., Gmernicki K.R., Long B.K. (2018). High Temperature, Living Polymerization of Ethylene by a Sterically-Demanding Nickel(II) α-Diimine Catalyst. Polymers.

[17] Wang F.-Z., Tian S.-S., Li R.-P., Li W.-M., Chen C.-L. (2018). Ligand steric effects on naphthyl-α-diimine nickel catalyzed α-olefin polymerization. Chin. J. Polym. Sci..

[18] Wang F., Chen C. (2019). A continuing legend: The Brookhart-type α-diimine nickel and palladium catalysts. Polym. Chem..

[19] Padilla-Vélez O., O’Connor K.S., Lapointe A.M., Macmillan S.N., Coates G.W. (2019). Switchable living nickel(ii) α-diimine catalyst for ethylene polymerisation. Chem. Commun..

[20] Hu X., Zhang Y., Zhang Y., Jian Z. (2020). Unsymmetrical Strategy Makes Significant Differences in α-Diimine Nickel and Palladium Catalyzed Ethylene (Co)Polymerizations. ChemCatChem.

[21] Muhammad Q., Pang W., Wang F., Tan C. (2020). Ortho-functionalized dibenzhydryl substituents in α-diimine Pd catalyzed ethylene polymerization and copolymeriza-tion. Polymers.

[22] Simon L.C., Patel H., Soares J.B.P., de Souza R.F. (2001). Polyethylene Made with In Situ Supported Ni-Diimine/SMAO: Replication Phenomenon and Effect of Polymerization Conditions on Polymer Microstructure and Morphology. Macromol. Chem. Phys..

[23] Preishuber-Pflugl P., Brookhart M. (2002). Highly Active Supported Nickel Diimine Catalysts for Polymerization of Ethylene. Macromolecules.

[24] Alobaidi F., Ye Z., Zhu S. (2003). Ethylene Polymerization with Silica-Supported Nickel-Diimine Catalyst: Effect of Support and Polymerization Conditions on Catalyst Activity and Polymer Properties. Macromol. Chem. Phys..

[25] Severn J.R., Chadwick J.C., Van Axel Castelli V. (2004). MgCl_2_-Based Supports for the Immobilization and Activation of Nickel Diimine Catalysts for Polymerization of Ethylene. Macromolecules.

[26] Huang R., Koning C.E., Chadwick J.C. (2007). Synergetic Effect of a Nickel Diimine in Ethylene Polymerization with Immobilized Fe-, Cr-, and Ti-Based Catalysts on MgCl_2_ Supports. Macromolecules.

[27] Schrekker H.S., Kotov V., Preishuber-Pflugl P., White P., Brookhart M. (2006). Efficient Slurry-Phase Homopolymerization of Ethylene to Branched Polyethylenes Using α-Diimine Nickel(II) Catalysts Covalently Linked to Silica Supports. Macromolecules.

[28] Li Y.-G., Pan L., Zheng Z.-J., Li Y.-S. (2008). Polymerization of ethylene to branched polyethylene with silica and Merrifield resin supported nickel(II) catalysts with α-diimine ligands. J. Mol. Catal. A Chem..

[29] Bahuleyan B.K., Oh J.M., Chandran D., Ha J.Y., Hur A.Y., Park D.-W., Ha C.S., Suh H., Kim I. (2010). Highly Efficient Supported Diimine Ni(II) and Iminopyridyl Fe(II) Catalysts for Ethylene Polymerizations. Top. Catal..

[30] Jiang H., He F., Wang H. (2008). A new strategy to prepare branching polyethylene by using an α-diimine nickel(II) complex covalently supported on MgCl_2_/AlRn(OEt)_3-n_. J. Polym. Res..

[31] Jiang H., Lu J., Wang F. (2010). Polymerization of ethylene using a nickel α-diimine complex covalently supported on SiO_2_–MgCl_2_ bisupport. Polym. Bull..

[32] Choi Y., Soares J.B.P. (2009). Synthesis of Supported Nickel Diimine Catalysts for Ethylene Slurry Polymerization. Macromol. Chem. Phys..

[33] Choi Y., Soares J.B. (2010). Ethylene slurry polymerization using nickel diimine catalysts covalently-attached onto MgCl_2_-based supports. Polymer.

[34] Huang C., Zakharov V.A., Semikolenova N.V., Matsko M.A., Mahmood Q., Talsi E.P., Sun W.-H. (2019). Comparisons between homogeneous and immobilized 1-(2,6-dibenzhydryl-4-nitrophenylimino)-2-mesityliminoacenaphthylnickel bromide as a precatalyst in ethylene polymerization. J. Catal..

[35] Zhang D., Jin G.-X. (2004). Radical co-polymerization of diiminedibromidenickel(II)-functionalized olefin with styrene: Synthesis of polymer-incorporated nickelII α-diimine catalysts for ethylene polymerization. Appl. Catal. A Gen..

[36] Jiang T. (2014). Preparation of spherical MgCl_2_/SiO_2_/THF-supported late-transition metal catalysts for ethylene polymerization. China Pet. Processing Petrochem. Technol..

[37] Bahuleyan B.K., Jermy B.R., Ahn I.Y., Suh H., Park D.-W., Ha C.S., Kim I. (2009). One-pot synthesis of spherical periodic mesoporous organosilica supported catalyst bearing Ni(II) α-diimine complexes for ethylene polymerization. Catal. Commun..

[38] Xu L., Ye Z., Cui Q., Gu Z., Mercier L. (2011). Surface-initiated catalytic ethylene polymerization within nano-channels of ordered mesoporous silicas for synthesis of hybrid silica composites containing covalently tethered polyethylene. Polymer.

[39] Favero C., Closs M.B., Galland G.B., Stieler R., Rossetto E., Bernardo-Gusmão K. (2019). A binary nickel diimine-MCM-41 supported catalyst and its application in ethylene polymerization. J. Catal..

[40] Zhang R.-F., Hou Y.-H., Wei X.-L., Zhao D.-D., Cui M.-M., Zhai F.-F., Li X.-L., Liu B.-Y., Yang M. (2020). Thermostable α-Diimine Nickel Complexes with Substituents on Acenaphthequinone-backbone for Ethylene Polymerization. Chin. J. Polym. Sci..

[41] Clas S.-D., Heyding R.D., McFaddin D.C., Russell K.E., Scammell-Bullock M.V., Kelusky E.C., St-Cyr D. (1988). Crystallinities of copolymers of ethylene and 1-alkenes. J. Polym. Sci. Pol. Phys..

[42] Galland G.B., de Souza R.F., Mauler R.S., Nunes F.F. (1999). ^13^C NMR Determination of the Composition of Linear Low-Density Polyethylene Obtained with [η^3^-Methallyl-nickel-diimine]PF_6_ Complex. Macromolecules.

[43] He X., Guo Y., Chen X., Wu B., Zou J., Wen Y., Chen D. (2021). Synthesis of MWNTs/SiO_2_ Supported Nickel and Palladium Complexes and their Application as Catalysts for Cyclic Olefins Polymerization. J. Organomet. Chem..

