# Supplementary material for: Slurry Homopolymerization of Ethylene Using Thermostable α-Diimine Nickel Catalysts Covalently Linked to Silica Supports via Substituents on Acenaphthequinone-Backbone
Source: Polymers (Basel). 2022 Sep 5;14(17):3684. doi: 10.3390/polym14173684 (PMC9459716; doi:10.3390/polym14173684)
Supplement: Supplementary file 1 [file polymers-14-03684-s001.zip › polymers-1834075-supplementary.pdf]

Electronic Supplementary Information

Slurry polymerization of ethylene using thermostable α-diimine nickel catalysts covalently linked to silica via substituents on acenaphthequinone-backbone

Kening Zong, Yanhui Hou, Xiaobei Zhao, Yali Sun, Binyuan Liu and Min Yang

Table of Contents

GPC curves of polyethylene S2

DSC curves of polyethylene S3

^{13}C NMR spectra of polyethylene S6

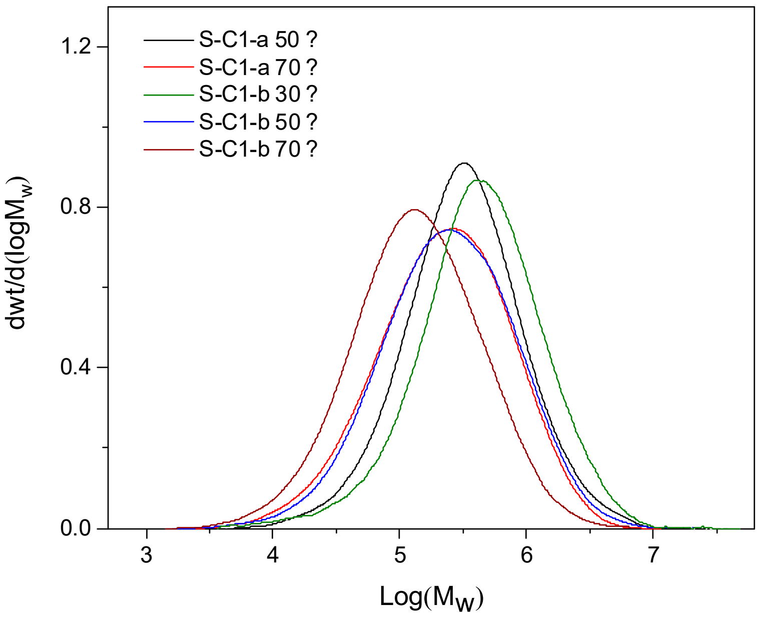

Figure S1. GPC curves of polyethylene obtained with S-C1-a/b (Entries 2, 3, 5, 6, 7 in Table 2)

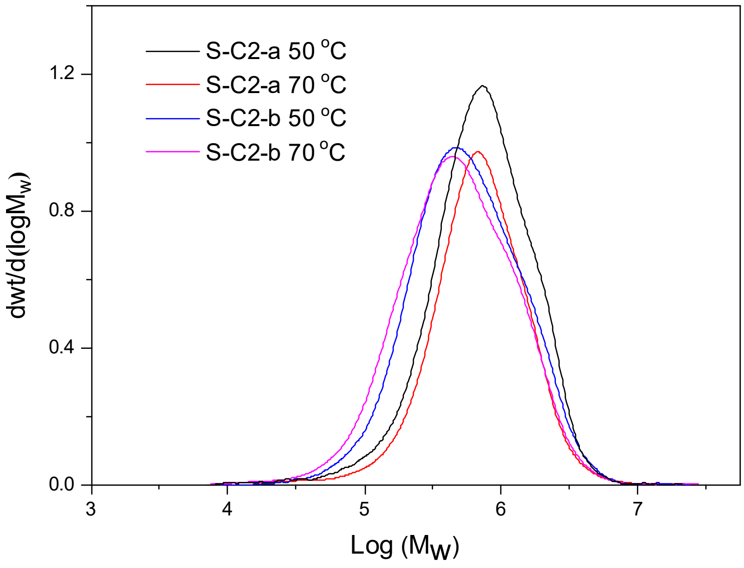

Figure S2. GPC curves of polyethylene obtained with S-C2-a/b (Entries 9, 10, 13, 14 in Table 2)

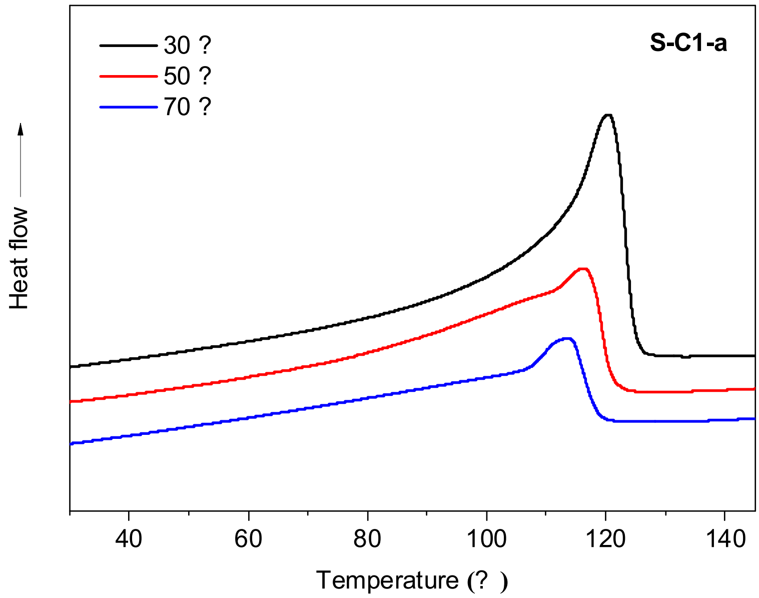

Figure S3. DSC curves of polyethylene obtained with S-C1-a (Entries 1- 3 in Table 2)

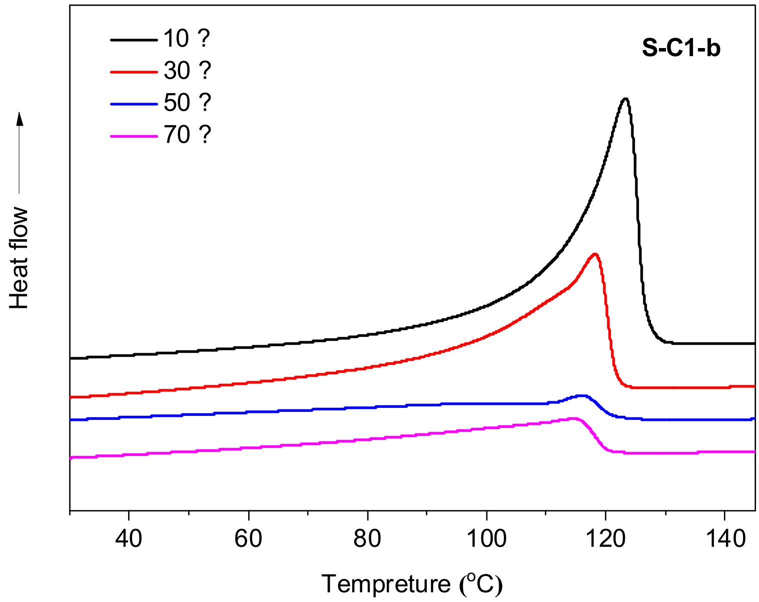

Figure S4. DSC curves of polyethylene obtained with S-C1-b (Entries 4-7 in Table 2)

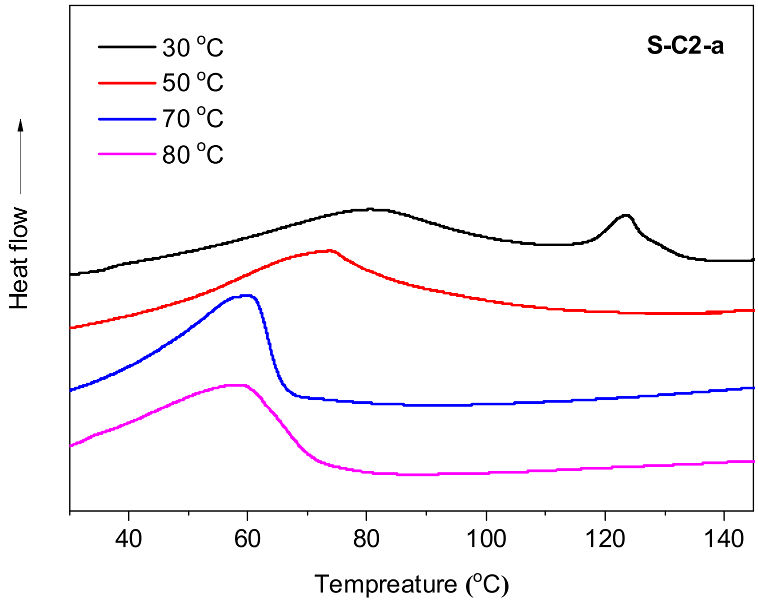

Figure S5. DSC curves of polyethylene obtained with S-C2-a (Entries 8-11 in Table 3)

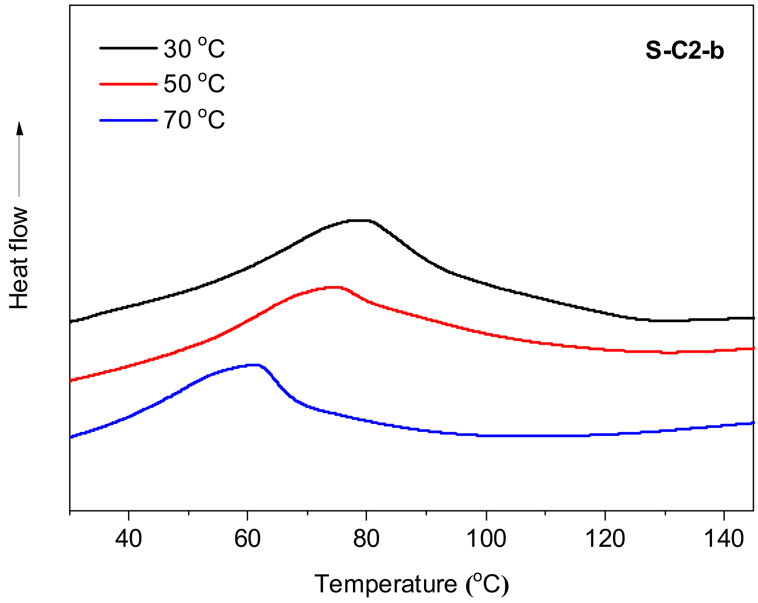

Figure S6. DSC curves of polyethylene obtained with S-C2-b (Entries 12-14 in Table 3)

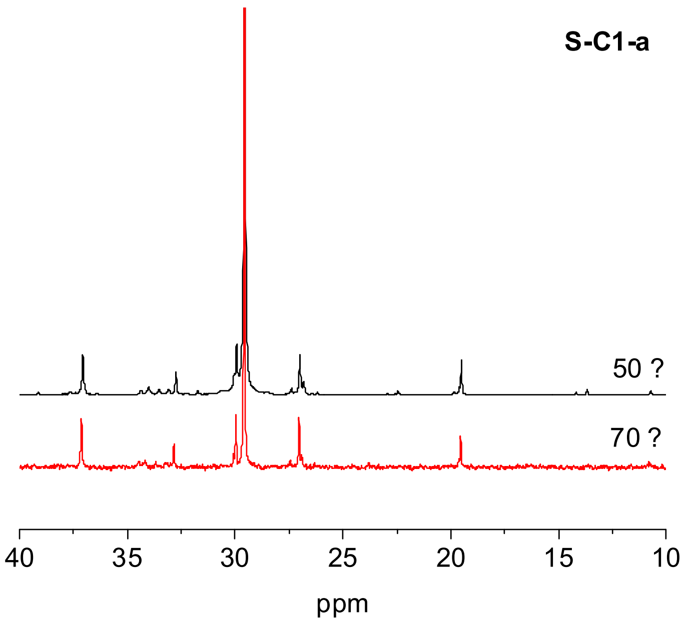

Figure S7. ^{13}**C NMR spectra of polyethylenes (Entries 2, 3 in Table 2).**

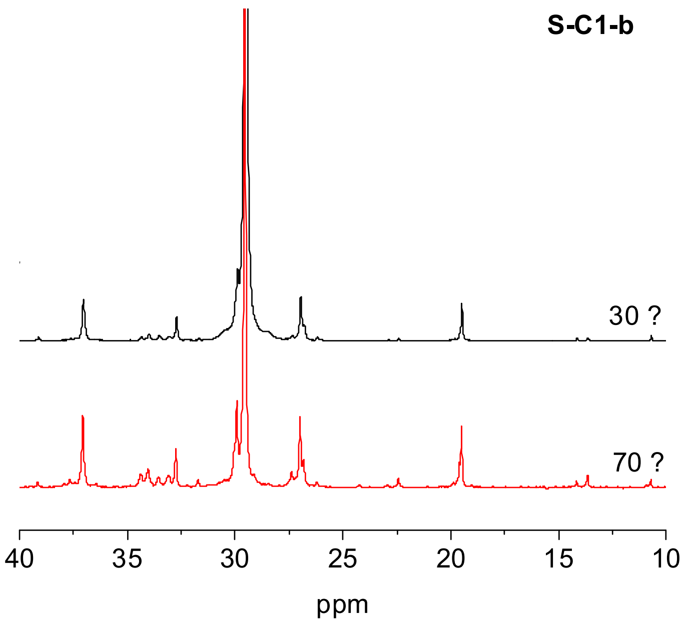

Figure S8. ^{13}**C NMR spectra of polyethylenes (Entries 5, 7 in Table 2).**

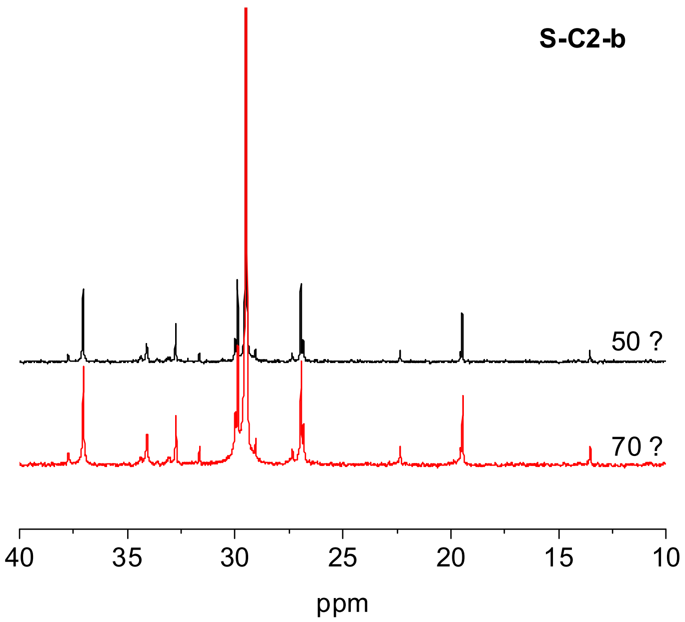

Figure S9. ^{13}C NMR spectra of polyethylenes (Entries 13, 14 in Table 3).
